# Cellular and extracellular matrix changes in anterior cruciate ligaments during human knee aging and osteoarthritis

**DOI:** 10.1186/ar4165

**Published:** 2013-02-14

**Authors:** Akihiko Hasegawa, Hiroyuki Nakahara, Mitsuo Kinoshita, Hiroshi Asahara, James Koziol, Martin K Lotz

**Affiliations:** 1 Department of Molecular and Experimental Medicine, The Scripps Research Institute, 10550 North Torrey Pines Road, La Jolla, CA 92037, USA; 2 Department of Orthopedic Surgery, Osaka Medical College, 2-7 Daigakumachi Takatsuki, Osaka 569-8686, Japan

## Abstract

**Introduction:**

Anterior cruciate ligament (ACL) degeneration is observed in most osteoarthritis (OA)-affected knee joints. However, the specific spatial and temporal relations of these changes and their association with extracellular matrix (ECM) degeneration are not well understood. The objective of this study was to characterize the patterns and relations of aging-related and OA-associated changes in ACL cells and the ECM.

**Methods:**

Human knee joints from 80 donors (age 23 through 94) were obtained at autopsy. ACL degeneration was assessed histologically by using a quantitative scoring system. Tissue sections were analyzed for cell density, cell organization, ECM components, ECM-degrading enzymes and markers of differentiation, proliferation, and stem cells.

**Results:**

Total cell number in normal ACL decreased with aging but increased in degenerated ACL, because of the formation of perivascular cell aggregates and islands of chondrocyte-like cells. Matrix metalloproteinase (MMP)-1, -3, and -13 expression was reduced in aging ACL but increased in degenerated ACL, mainly in the chondrocyte-like cells. Collagen I was expressed throughout normal and degenerated ACL. Collagen II and X were detected only in the areas with chondroid metaplasia, which also expressed collagen III. Sox9, Runt-related transcription factor 2 (Runx2), and scleraxis expression was increased in the chondrocyte-like cells in degenerated ACL. Alpha-smooth muscle actin (α-SMA), a marker of myofibroblasts and the progenitor cell marker STRO-1, decreased with aging in normal ACL. In degenerated ACL, the new cell aggregates were positive for α-SMA and STRO-1.

**Conclusions:**

ACL aging is characterized by reduced cell density and activation. In contrast, ACL degeneration is associated with cell recruitment or proliferation, including progenitor cells or myofibroblasts. Abnormally differentiated chondrocyte-like cell aggregates in degenerated ACL produce abnormal ECM and may predispose to mechanical failure.

## Introduction

Anterior cruciate ligament (ACL) degeneration is observed in most osteoarthritis (OA)-affected knee joints [1,2]. Our recent observations indicate that histologic changes in ACL, in particular chondroid metaplasia, collagen fiber disorganization, and mucoid degeneration, can occur before the onset of significant cartilage degeneration in individuals without a history of knee trauma and at a relatively young age [1]. It is thus possible that changes in ACL cells and extracellular matrix (ECM) can be driven by extrinsic factors, for example, in knees with cartilage damage and inflammation or, alternatively, intrinsic primary changes in ACL cells and ECM may lead to ACL mechanical failure.

Limited information is available on changes in ACL cell density. Cell density in human ACL has been reported to decrease with age [3], but cell density can also be increased in ACLs from old OA patients [4]. Changes of ACL cell activation and differentiation status have also been documented. Chondroid metaplasia is a feature of degenerated ACL [2,5-7] and may precede and predispose to structural failure [3,8].

ACLs contain a small subpopulation of fibroblasts positive for α-smooth muscle actin (α-SMA) [9], as well as cells that express certain markers of mesenchymal progenitor cells, including nucleostemin, SSEA-4, STRO-1, and Oct-4 [10-12].

The ECM of ACL is composed of collagen types I, II, III, and V, elastin, and proteoglycans [13,14], with type I collagen being the main determinant of tensile strength [15]. Ruptured ACLs express much higher quantities of mRNA for type I collagen and type III collagen, and higher quantities of biglycan than normal ACL [16,17].

Collectively, this prior work demonstrated that ACL aging and degeneration are associated with ACL cell death, cell proliferation or recruitment, and changes in ACL differentiation and activation. However, the specific spatial and temporal relations of these changes and their association with ECM degeneration are not well understood. The objective of this study was to characterize cellular and ECM changes in human ACL in aging and degeneration from a large number of donors across the entire adult age spectrum at all stages of OA development.

## Materials and methods

### Tissue procurement

Human knee joints were processed within 72 hours after death. In this study, 150 human knee joints were analyzed, including 41 male and 39 female donors, with a mean age of 65.5 ± 19.0 years (range, 23 through 94 years). From 70 of 80 donors, we obtained both knees, whereas from 10 of 80 donors, we obtained only one knee. Subjects with a history of knee trauma or surgery were excluded. The causes of death and comorbidities were similar to those of the general population in the United States. Human knee joints were obtained at autopsy with approval of the Scripps Human Subjects Committee. All study donors provided written informed consent, according to the Declaration of Helsinki.

### Morphologic analysis of articular cartilage

All cartilage surfaces (femoral condyles, trochlea, and tibial plateaus) were graded macroscopically by using a modified Outerbridge scoring system and the International Cartilage Repair Society (ICRS) knee map, as described previously [1].

### Macroscopic and histologic analysis of ACL

ACL degeneration was assessed both macroscopically and histologically, as described [1], by using a modification of a previously reported scoring system [2]. Macroscopic assessment showed that ACLs in 91 knees were normal, 46 were abnormal, and 13 were ruptured. The following categories were examined and scored for each ligament: (a) inflammation in the ACL substance, (b) mucoid degeneration, (c) chondroid metaplasia, (d) cystic changes, and (e) orientation of collagen fibers. Each category was scored from 0 to 3 for the severity of the degenerative change on both transverse and longitudinal sections, and a single score was assigned for each ACL. The highest summed score of ligament degeneration (total ACL score) was 15 if all five histologic categories were scored 3 (severe).

ACL degeneration was graded as normal, mild, moderate, and severe after considering the total ACL substance score. Degeneration of ACL was considered normal if the total ACL score was 0 to 1, mild if it was 1.5 to 5, moderate if it was 5.5 to 10, and severe if it was higher than 10. If orientation of collagen fibers, mucoid degeneration, and/or cystic changes were scored 3, or ACL was completely degraded, and only a few remnants were detectable macroscopically, it was considered severe. Donor distribution among the different ACL grades was as follows: normal (*n *= 20), mild (*n *= 92), moderate (*n *= 22), and severe (*n *= 16).

To characterize ACL cellular and ECM changes in aging and degeneration, the donor population was divided into three groups; normal (<45 years old with normal ACL and normal cartilage), aging (>60 years old with mild ACL degeneration and minimal cartilage degeneration), and degenerated (>60 years old with moderate to severe ACL degeneration and cartilage degeneration). ACL sample distribution among the three different groups was as follows: normal (*n *= 10 from 7 donors), aging (*n *= 37 from 23 donors), and degenerated (*n *= 27 from 19 donors). Then 73 ACLs from 47 donors were excluded from the present study because they did not match the criteria. Three ACLs from two donors were completely degraded, and ACL samples were not collected.

### Immunohistochemistry

For each group, as defined earlier, 6 normal, 12 aging, and 10 degenerated ACL samples were used for immunostaining (Table 1). Paraffin-embedded ACL samples were first deparaffinized in xylene substitute Pro-Par Clearant (Anatech, Battle Creek, MI, USA) and ethanol before rehydration in water. After a wash with phosphate-buffered saline (PBS), antigen retrieval was performed by incubation with trypsin for 30 minutes at 30°C before applying type I collagen, type II collagen, type X collagen, Sox9, Runx2, Scleraxis, and α-SMA antibody, or hyaluronidase for 60 minutes at 37°C before applying type III collagen and aggrecan antibody. Endogenous peroxidase was quenched for 10 minutes with 3% H_2_O_2 _in methanol. Sections were blocked with 5% normal rabbit serum for MMP-3 or 10% normal goat serum for other antibodies for 30 minutes at room temperature. Sections were incubated overnight at 4°C with primary antibodies against MMP-1 (mab901, R&D Systems, Minneapolis, MN, USA; 2 μg/ml), MMP-3 (sc-6839, Santa Cruz Biotechnology, Santa Cruz, CA, USA; 1:100 dilution), MMP-13 (MAB3321, Chemicon International, Temecula, CA, USA; 1:1,000 dilution), Sox9 (AB5535, Chemicon International, Temecula, CA, USA; 2 μg/ml), Runx2 (sc-10758, Santa Cruz Biotechnology, Santa Cruz, CA, USA; 1:50 dilution), Scleraxis (ab58655, Abcam, Cambridge, MA, USA; 5 μg/ml), Ki-67 (ab15580, Abcam; 1:200 dilution), α-SMA (ab5694, Abcam, Cambridge, MA, USA; 1:300 dilution), STRO-1 (mab1038, R&D Systems, Minneapolis, MN, USA; 0.5 μg/ml), collagen I (ab292, Abcam, Cambridge, MA, USA; 1 μg/ml), collagen II (II-II6B3, Hybridoma Bank, Iowa City, IA; 2 μg/ml), collagen III (AP07843PU-N, Acris Antibodies, San Diego, CA, USA; 2 μg/ml), collagen X (ab49945, Abcam, Cambridge, MA, USA; 1:2,000 dilution), and aggrecan (ab3773, Abcam, Cambridge, MA, USA; 1:40 dilution), or negative controls (normal goat IgG, rabbit IgG, mouse IgG or IgM; 1 μg/ml).

**Table 1:** Donor information

Cartilage grade	Normal	Aging	Degenerated
Number	6 ACLs from 4 donors	12 ACLs from 9 donors	10 ACLs from 7 donors
Female/male	3:1	5:4	3:4
Age, mean ± SD years	33.3 ± 11.3 (23-44)	75.0 ± 11.6 (60-94)	78.9 ± 12.3 (60-92)
Body mass index, mean ± SD kg/m2	23.6 ± 6.7 (15.9-29.2)	23.4 ± 5.6 (16.8-33.6)	26.0 ± 8.2 (19.1-43.8)

After washing with PBS, sections were incubated with biotinylated goat anti-mouse IgM (Jackson ImmunoResearch Laboratories, West Baltimore, PA, USA; 1:500 dilution) for STRO-1 and collagen type X, biotinylated goat anti-mouse secondary antibody (Vector Laboratories, Burlingame, CA, USA; 1:200 dilution) for MMP-13, collagen type II and aggrecan, biotinylated rabbit anti-goat secondary antibody (Vector Laboratories, Burlingame, CA, USA; 1:200 dilution) for MMP-3 or biotinylated goat anti-rabbit secondary antibody (Vector Laboratories, Burlingame, CA, USA; 1:200 dilution) for collagen type I, collagen III, Sox9, Runx2, Scleraxis, and α-SMA for 30 minutes and then incubated with Vectastain ABC-AP kit (AK-5000; Vector Laboratories, Burlingame, CA, USA) for 30 minutes at room temperature.

Finally, sections were stained with an alkaline phosphatase substrate kit (Vector Laboratories, Burlingame, CA, USA). The slides were rinsed in tap water and counterstained with hematoxylin. Slides were washed and mounted with Prolong Gold Antifade Reagent (Invitrogen, Carlsbad, CA, USA).

### Quantification and localization of positive cells in human ACLs

For the quantification of total cell number and positive cells for each marker in the ACL, at least three different macroscopic fields (40× magnification) per each sample, showing the ACL mid-substance, were randomly chosen. The insertion sites were not included in the analysis. The total cell number and total number of positive cells for each marker was counted in each histologic zone by two different readers, and the percentage of positive cells was calculated in each field. The interclass correlation between two readers was 0.88. The mean of the percentage of positive cells by two different readers was used for the statistical analysis.

### Western blotting

Human ACL cells were isolated from the midsubstance of ACLs and cultured with Dulbecco modified Eagle medium (DMEM) supplemented with 10% fetal bovine serum (FBS) (Invitrogen, Tastrup, Denmark) and 1% penicillin/streptomycin (Invitrogen). ACL cells were used at passage 1 (P1). Western blotting was performed with the LiCor immunofluorescence detection system (LI-COR Biosciences, Lincoln, NE, USA). In total, 20 μg of each cell lysate was separated on 4% to 20% SDS PAGE gels and transferred to nitrocellulose membranes. As primary antibodies we used, mouse anti-MMP-1 antibody (mab901; R&D Systems, Minneapolis, MN, USA; 1 μg/ml), goat anti-MMP-3 antibody (sc-6839, Santa Cruz Biotechnology, Santa Cruz, CA, USA; 1:1,000 dilution), or rabbit anti-MMP-13 antibody (ab75606, Abcam, Cambridge, MA, USA; 1:500 dilution) and rabbit anti-GAPDH (14C10, Cell Signaling, Danvers, MA; 1:2,000 dilution) or mouse anti-GAPDH antibody (AM4300, Ambion, Austin, TX, USA; 1:5,000 dilution). As secondary antibodies, we used goat anti-mouse-IRDye 800 (LI-COR Biosciences, Lincoln, NE USA; 1/10,000), donkey anti-goat IRDye 800 (LI-COR Biosciences, Lincoln, NE USA; 1/15,000), or goat anti-rabbit-IRDye 800 (LI-COR Biosciences, Lincoln, NE USA; 1/10,000) for MMPs and goat anti-rabbit-IRDye 680 (LI-COR Biosciences, Lincoln, NE USA; 1/10,000) or goat anti-mouse-IRDye 680 (LI-COR Biosciences, Lincoln, NE USA; 1/10,000) for GAPDH. Images were acquired on the LiCor Odyssey.

### Statistical analysis

The results are presented as mean ± SEM (standard error of the mean). Differences among groups were determined with one-way analysis of variance (ANOVA) or Kruskal-Wallis (nonparametric one-way analysis of variance) procedures with continuous variables, and χ^2 ^procedures with dichotomous variables. Pairwise group comparisons subsequent to the overall ANOVA tests were undertaken with the Tukey Student range procedure after parametric ANOVA, or, with the Dwass-Steel-Critchlow-Fligner (DSCF) procedure, after Kruskal-Wallis. The *P *values of <0.05 were considered statistically significant.

## Results

### Cell density and cell arrangements

**Figure 1:**
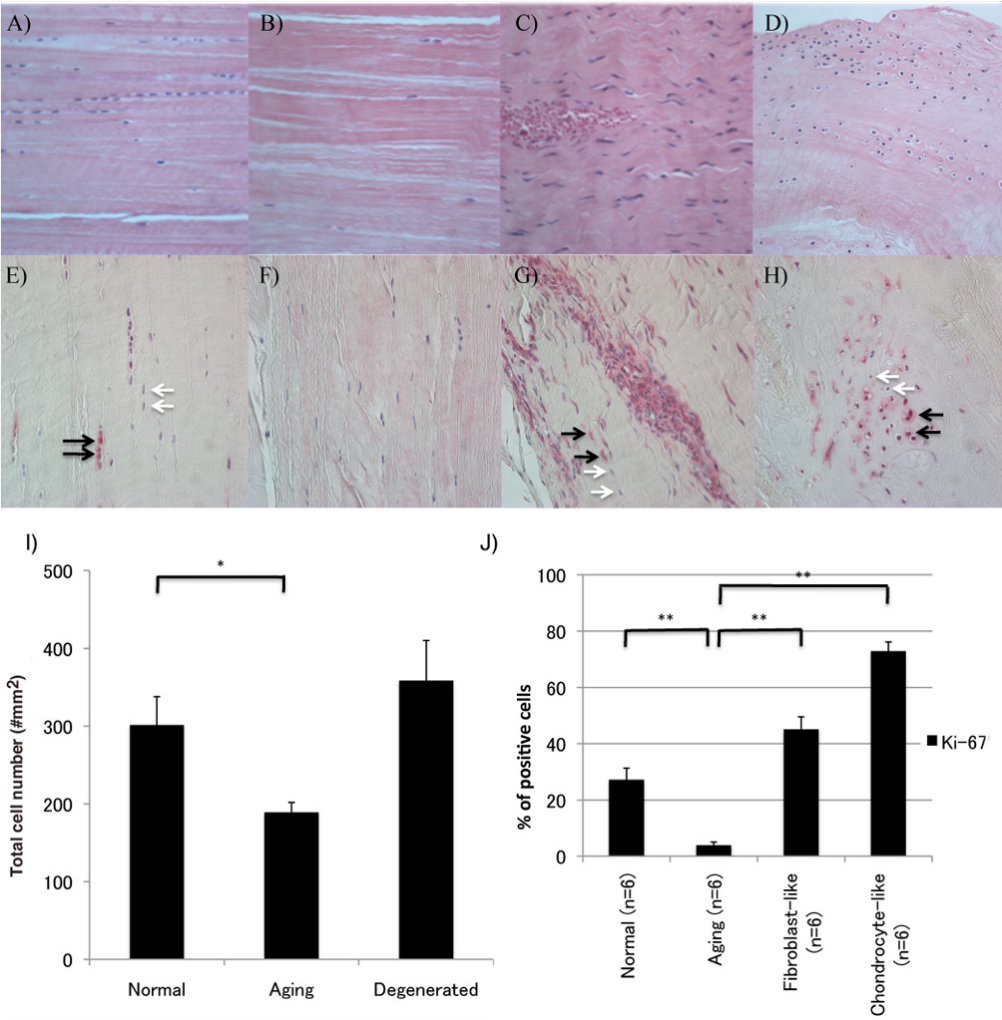
**Cell organization, cell density, and Ki-67 expression in ACL**. **(A-D) **H&E staining; **(E-H) **Ki-67 (original magnification ×40). Sections of ACL representing **(A, E) **normal cell distribution in ACL from young normal knee; **(B, F) **hypocellular ACL from aging knee; **(C, G) **fibroblast-like cell aggregates in degenerated ACL; **(D, H) **chondrocyte-like cell aggregates in degenerated ACL. Black arrows, Ki-67-positive cells; white arrows, Ki-67-negative cells. The graph **(I) **represents the total cell density in each ACL group. The graph **(J) **represents the percentage of Ki-67-positive cells. The results are presented as mean ± SEM. *P < 0.05; **P < 0.01.

The cell density in histologically normal ACLs from young donors (<45 years old) with normal (Grade 0) knee cartilage was 301.4 ± 36.6/mm^2^, and this was significantly reduced to 188.9 ± 13.0/mm^2 ^in ACL from old donors (>60 years old), with minimal changes in the articular cartilage (Grade I cartilage) (*P *= 0.023) (Figure 1A, B, I). In contrast, cell density in degenerated ACLs from old donors (>60 years old) with degenerated cartilage (Grade II to IV) (358.3 ± 51.8/mm^2^) was as high as that in the ACL from young donors (Figure 1A, C, D).

In older donors with grade I cartilage, an overall reduction in cell density was seen across the midsubstance of the ACL (Figure 1B). The ACL from older donors with cartilage degeneration (Grades II through IV) showed also a reduction in normally arranged cells. However, hypercellular areas were observed around blood vessels, and these cells had a fibroblast-like shape (Figure 1C). A separate type of new cell aggregates occurred independent of blood vessels and included cells with a chondrocyte-like morphology (Figure 1D). These cells were not aligned with collagen fibers, and cell arrangements represented islands with large numbers of cells interspersed within the hypocellular areas. Fibroblast-like cell aggregates were present in 12 (44.4%) of 27 degenerated ACL groups, and this was significantly more common than normal (0 of 8; none) and aging group (5 (13.5%) of 37) (*P *= 0.004 with χ^2 ^analysis). Chondrocyte-like cell aggregates were present in 14 (51.9%) of 27 of the degenerated ACL group, and this was significantly more common than in the normal (none of eight) and aging group (five (13.5%) of 37 (*P *= 0.0005 by χ^2 ^analysis). Nine (33.3%) of 27 degenerated ACLs contained both fibroblast-like cell aggregates and chondrocyte-like cell aggregates. Dense cell clusters, as typically seen in OA articular cartilage, were observed in 4 of 150 ACLs (2.7%), which were associated with disruption of collagen fibers and/or mucoid degeneration from knees with cartilage degeneration.

Thus, a general reduction in ACL cellularity occurs in aging. However, in degenerated ACLs, two distinct types of localized high cell-density aggregates are noted. In the following immunohistochemical studies, fibroblast-like cell aggregates and chondrocyte-like cell aggregates were examined separately.

### Cell proliferation

Ki-67 is a cell-proliferation marker [5]. In normal ACLs, 27.2% ± 4.1% of ligament cells were Ki-67 positive. The number of Ki-67-positive cells decreased with aging (3.9% ± 1.2%; *P *< 0.0001) (Figure 1E, F), whereas in degenerated ACLs, the number of Ki-67-positive cells increased (59.0% ± 3.6%; *P *< 0.0001). Of fibroblast-like cells, 45.1% ± 4.5% were Ki-67 positive, whereas 72.9% ± 3.3% of chondrocyte-like cells were Ki-67 positive (Figure 1G, H).

### Cell differentiation

**Figure 2:**
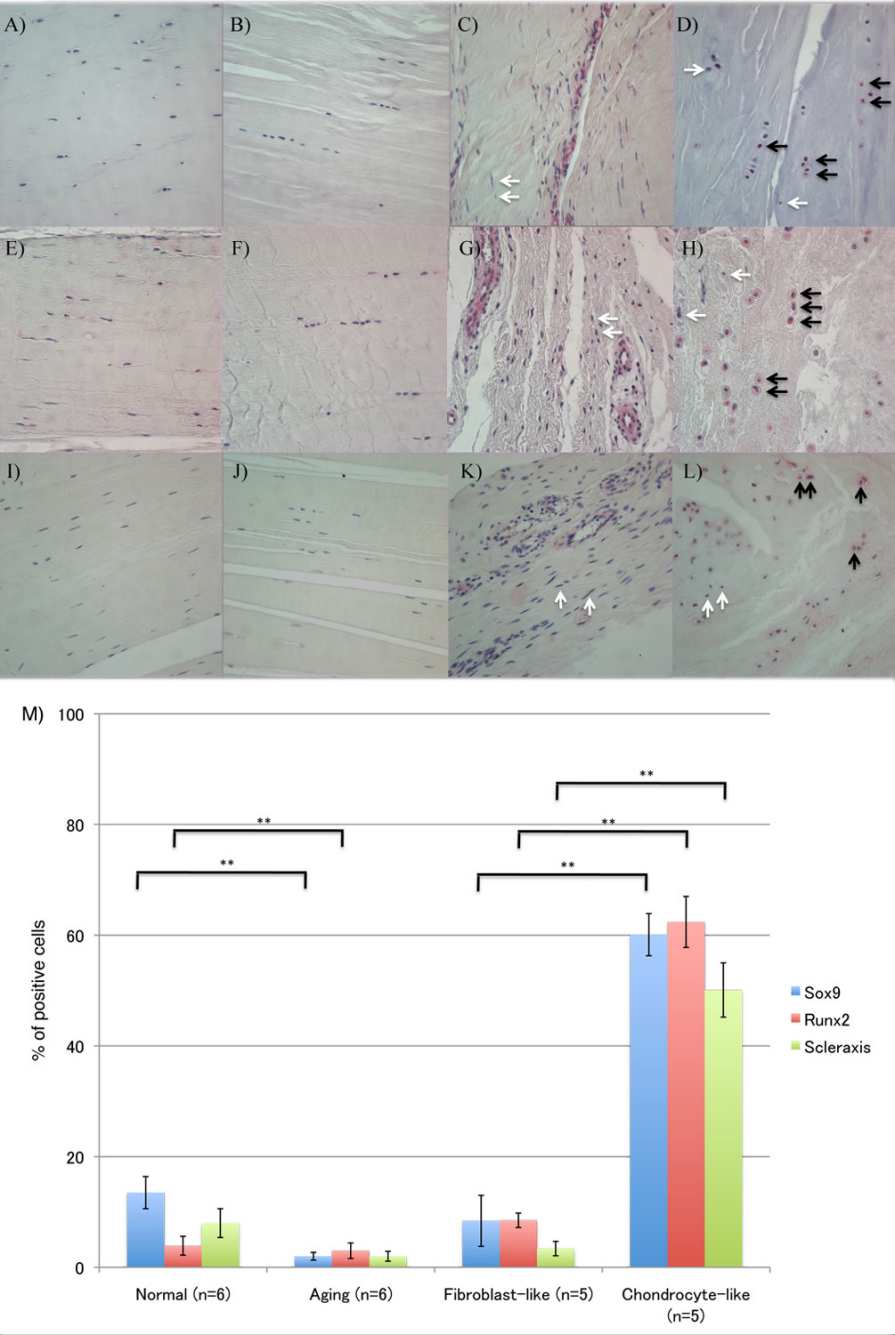
**Sox9, Runx2, and Scleraxis expression**. **(A-D) **Sox9; **(E-H) **Runx2; **(I-L) **Scleraxis. **(A,E,I) **ACL from normal knee; **(B,F,J) **ACL from aging knee; (**C,G,K) **fibroblast-like cell aggregates in the degenerated ACL. **(D,H,L) **Chondrocyte-like cell aggregates in the degenerated ACL. Black arrows, positive cells; white arrows, negative cells. (Original magnification ×40). The graph **(M) **represents the percentage (mean ± SEM) of Sox9-, Runx2-, and Scleraxis-positive cells. **P < 0.01.

Sox9 is a transcription factor known to be involved in the modulation of the chondrocyte phenotype [18]. In normal ACLs, 13.5% of cells were Sox9 positive, and Sox9-positive cells decreased with aging (2.0% ± 0.7%; *P *< 0.0001). In the degenerated ACLs, 60.1% of chondrocyte-like cells were Sox9 positive; however, only 8.4% of fibroblast-like cells were Sox9 positive (*P *< 0.0001) (Figure 2A through D).

Runx2 is a transcription factor that regulates chondrocyte hypertrophy [18]. In normal and aging ACLs, a few Runx2-positive cells were found. In the degenerated ACLs, 8.5% of fibroblast-like cells were Runx2 positive, whereas 62.4% of chondrocyte-like cells were Runx2 positive (*P *< 0.0001) (Figure 2E through H).

Transcription factor scleraxis (Scx) is a highly specific marker of the tendon/ligament lineage that regulates fibroblast differentiation and ECM synthesis during embryonic tendon/ligament development [19,20]. In normal ACLs, 8.0% of cells were Scx positive, and the percentage of Scx-positive cells in the aging group was 2.0% ± 0.9% (*P *= 0.09) (Figure 2I, J). In the degenerated ACLs, 50.1% of chondrocyte-like cells were Scx positive; however, only 3.4% of fibroblast-like cells were positive (*P *< 0.0001) (Figure 2K, L). These results indicate abnormal expression of differentiation markers in chondrocyte-like aggregates in the degenerated ACL.

### Progenitor cell markers

**Figure 3:**
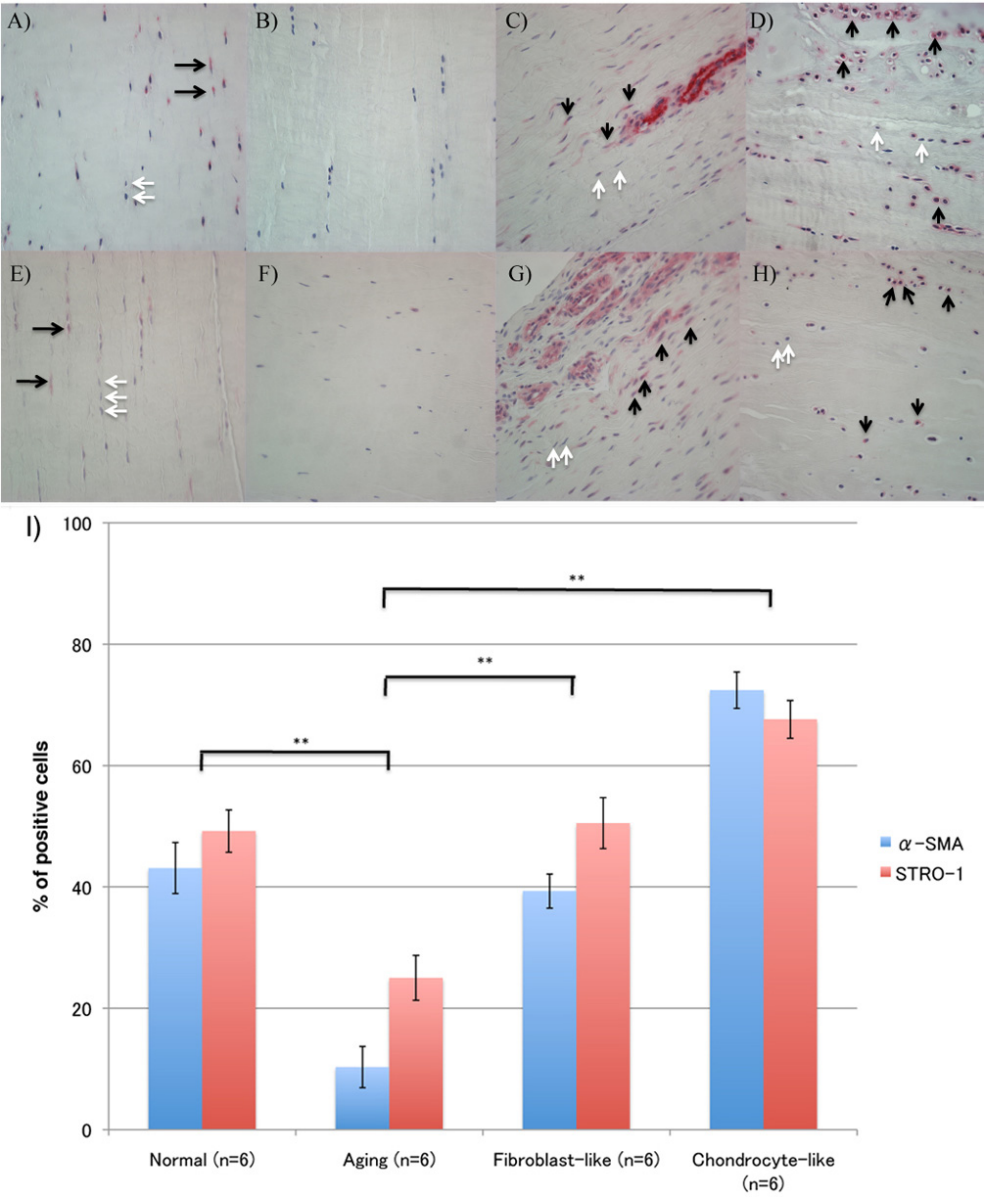
**α-SMA and STRO-1 expression**. **(A-D) **α-SMA; **(E-H) **STRO-1. **(A,E) **ACL from young normal knee; **(B,F) **ACL from aging knee; **(C,G) **fibroblast-like cell aggregates in the degenerated ACL; **(D,H) **chondrocyte-like cell aggregates in the degenerated ACL. Black arrows, positive cells; white arrows, negative cells. (Original magnification ×40). **(I) **The graph represents the percentage (mean ± SEM) of α-SMA- and STRO-1-positive cells. **P < 0.01.

α-Smooth muscle actin (α-SMA) is a marker of myofibroblasts and also immature or stem cells [21]. α-SMA-positive cells were observed in dense collagenous tissue, perivascular area, and lining cells in the synovial sheath. In normal ACLs, 43.1% of ligament cells were positive (Figure 3A). The number of positive cells decreased with aging (10.3% ± 3.4%; *P *< 0.0001) (Figure 3B). But the average percentage of α-SMA positive cells in degenerated ACLs (55.9 ± 3.6%) was higher than that in the aging group (10.3% ± 3.4%; *P *< 0.0001). 72.4% of chondrocyte-like cells and 39.4% of fibroblast-like cell aggregates were α-SMA positive (Figure 3C, D).

STRO-1 is a marker of mesenchymal stem cells [22]. STRO-1-positive cells were observed in dense collagenous tissue, perivascular areas, and lining cells in the synovial sheath. In normal ACLs, 49.2% ± 3.5% of ligament cells were STRO-1 positive (Figure 3E). The number of positive cells decreased with aging (25.0% ± 3.7%; *P *< 0.0001) (Figure 3F), but the average percentage of STRO-1-positive cells in ACLs from the degenerated group (59.0% ± 3.0%) was higher than that in the aging group (25.0% ± 3.7%; *P *< 0.0001); 67.6% ± 3.1% of chondrocyte-like cells were positive, whereas 50.5% ± 4.2% of fibroblast-like cells were STRO-1 positive (Figure 3G, H). The expression patterns of α-SMA and STRO-1 in normal and degenerated ACLs were similar (Figure 3I).

### MMP-1, MMP-3, and MMP-13 expression

**Figure 4:**
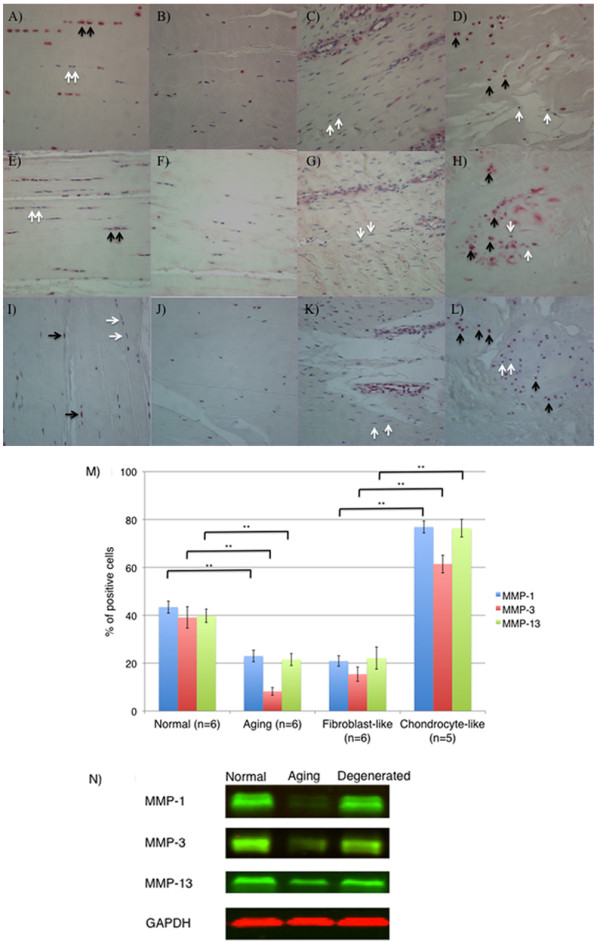
**MMP-1, MMP-3, and MMP-13 expression**. **(A-D) **MMP-1; **(E-H) **MMP-3; **(I-L) **MMP-13 **(A,E,I) **ACL from young normal knee; **(B,F,J) **ACL from aging knee; **(C,G,K) **fibroblast-like cell aggregates in the degenerated ACL; **(D,H,L) **chondrocyte-like cell aggregates in the degenerated ACL. (Original magnification ×40). The graph **(M) **represents the percentage (mean ± SEM) of MMP-1-, MMP-3-, and MMP-13-positive cells in each group. **P < 0.01. **(N) **Western blotting for MMP-1, MMP-3, and MMP-13 (*n *= 3 for each category).

The average percentage of MMP-1-positive cells in the aging group (23.0% ± 2.3%) was significantly lower than in the normal and degenerated groups (43.4% ± 2.5% and 51.5% ± 5.1; *P *= 0.0003; *P *= 0.015, respectively) (Figure 4A, B, M). The average percentage of MMP-1-positive cells in ACLs from the degenerated group, however, was as high as that in the young group. In regard to the two different patterns of cell aggregates in the degenerated ACLs, only 20.9% of the fibroblast-like cells near the blood vessels were MMP-1 positive, whereas 76.9% of chondrocyte-like cells were MMP-1 positive (*P *< 0.0001) (Figure 4C, D, M).

The average percentage of MMP-3-positive cells in the aging group (8.2% ± 1.6%) was significantly lower than in the normal and degenerated group (39.1% ± 4.5% and 36.3% ± 4.7; *P *= 0.0002; *P *= 0.0009, respectively) (Figure 4E, F, M). But the average percentage of MMP-3-positive cells in ACLs from the degenerated group was as high as in the young group. In regard to the two different patterns of cell aggregates in the degenerated ACLs, only 15.4% of the fibroblast-like cells near the blood vessels were MMP-3 positive, whereas 65.4% of chondrocyte-like cells were MMP-3 positive (*P *< 0.0001) (Figure 4G, H, M).

The average percentage of MMP-13-positive cells in normal ACL (39.8% ± 2.8%) also decreased with aging (21.5% ± 2.5%; *P *= 0.0002) (Figure 4I, J, M). In the degenerated ACL, 21.2% of the fibroblast-like cells near the blood vessels were MMP-13 positive, whereas 76.4% of chondrocyte-like cells were MMP-13 positive (*P *< 0.0001) (Figure 4K, L, M). We used Western blotting to quantify MMP-1, -3, and -13. The expression of MMP-1, -3, and -13 decreased with aging. However, the expression of MMP-1, -3, and -13 in the degenerated group was as high as in the young-normal group (Figure 4N).

Thus, constitutive MMPs expression declines with aging, and a marked upregulation occurs in degenerated ACLs in the joints with cartilage degeneration.

### Extracellular matrix changes

**Figure 5:**
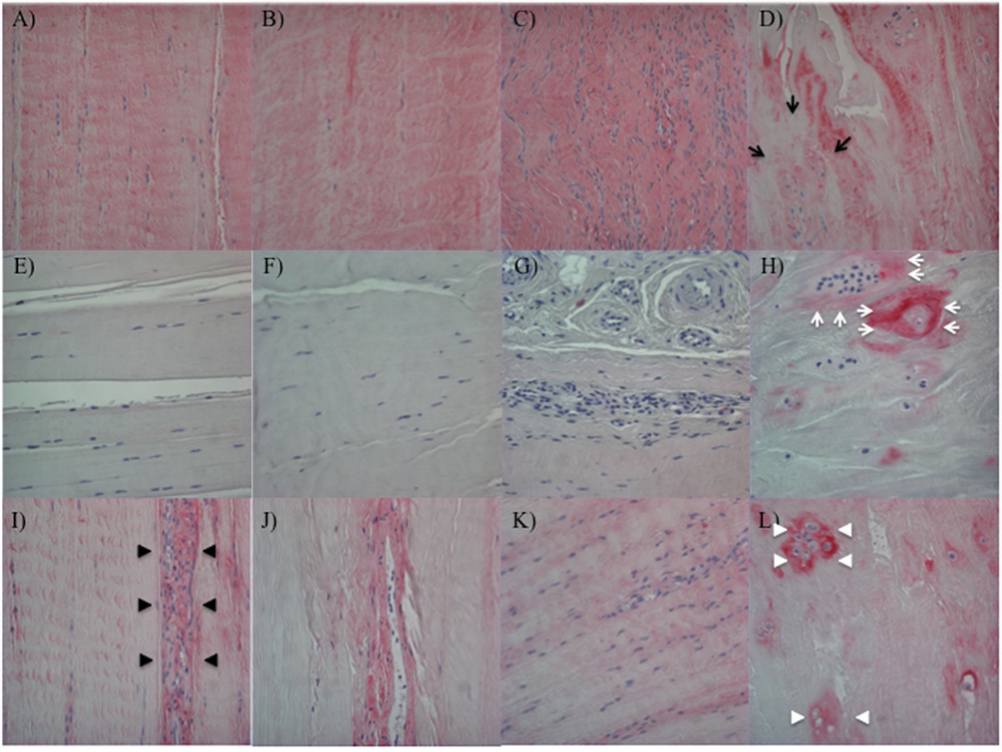
**Collagen types I, II, and III**. **(A-D) **Collagen type I, **(E-H) **collagen type II, **(I-L) **collagen type III **(A, E, I) **ACL from young normal knee; **(B, F, J) **ACL from aging knee; **(C, G, K) **fibroblast-like cell aggregates in the degenerated ACL; **(D, H, L) **chondrocyte-like cell aggregates in the degenerated ACL. Most collagen bundles were type I collagen positive **(A-D)**, however, staining intensity of the ECM around chondrocyte-like cell aggregates was lower (black arrows). Type II collagen-positive area is observed around chondrocyte-like cell aggregates (white arrows). In the normal ACL, type III collagen is located within the loose connective tissue (black arrowheads) that divides the collagen fibrils of the ligament into small bundles but not dense collagenous tissues. In the degenerated ACL, type III collagen (white arrowheads). (Original magnification ×40).

To assess ECM changes, we performed immunohistochemistry for collagens type I, II, III, and X and aggrecan. In the normal and aging ACL, most collagen bundles were type I collagen positive (Figure 5A, B). In the degenerated ACL, ECM around fibroblast-like cell aggregates were type I collagen positive; however, staining intensity of ECM around chondrocyte-like cell aggregates was lower (Figure 5C, D).

In the normal ACL, type II collagen is located in fibrocartilaginous zones, situated where the ligament inserts into bone [14]. Figure 5E and 5F shows histologic sections from the ACL midsubstance, and these regions were negative. In the degenerated ACL with chondroid metaplasia, we observed type II collagen around the chondrocyte-like cells in the ACL midsubstance but not the fibroblast-like cells (Figure 5E through H).

**Figure 6:**
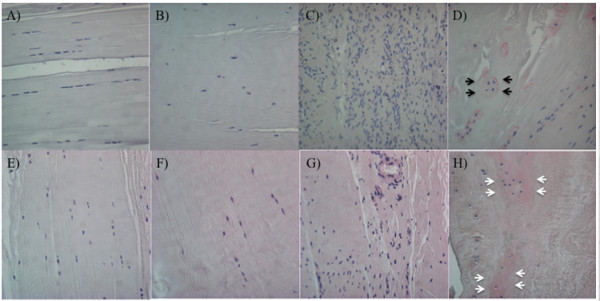
**Collagen type X and aggrecan**. **(A-D) **Collagen type X; **(E-H) **aggrecan; **(A,E) **ACL from young normal knee; **(B,F) **ACL from aging knee; **(C,G) **fibroblast-like cell aggregates in the degenerated ACL; **(D,H) **chondrocyte-like cell aggregates in the degenerated ACL. Type X collagen-positive area is observed around chondrocyte-like cell aggregates (black arrows), and aggrecan was observed around chondrocyte-like cell aggregates in the ACL midsubstance (white arrows). (Original magnification ×40).

In the normal ACL, type III collagen is located within the loose connective tissue that divides the collagen fibrils of the ligament into small bundles but not dense collagenous tissues. In the degenerated ACL, we observed type III collagen in the ACL midsubstance with chondrocyte-like cell aggregates as well as the loose connective tissues (Figure 5I through L). Type X collagen is a hypertrophic chondrocyte marker. We observed type X collagen around the chondrocyte-like cells but not the fibroblast-like cells (Figure 6A through D). Aggrecan is expressed predominantly in fibrocartilaginous zone of normal tendon and ligament [23]. Aggrecan was not observed within the ligament midsubstance. But in the ACL with chondroid metaplasia, aggrecan was also observed around the chondrocyte-like cells within the ligament midsubstance. In degenerated ACL, chondrocyte-like cells were aggrecan positive, whereas fibroblast-like cells were negative (Figure 6E through H).

## Discussion

ACL rupture represents a risk factor for cartilage degradation and OA development [24,25]. Aging-related degenerative changes in the ACL may also contribute to OA onset and progression, but mechanisms of ACL aging and degradation are not well characterized [26,27]. The present study used a large collection of human knee joints to document changes in ACL cells and extracellular matrix and correlated these changes with overall ACL histopathology grade and macroscopic assessment of the articular surfaces.

To profile normal ACL aging, we analyzed ACLs from a subset of older individuals with macroscopically normal ACLs and minimal degeneration of articular cartilage. The ACLs in these knees differed from normal ACLs from younger donors by an overall reduction in tissue cellularity and reduced cell activation and proliferation.

Degenerated ACLs showed marked changes in cell organization. As compared with normal aging ACLs, a further reduction occurred in the cells that are normally distributed along collagen bundles. However, total cell numbers were increased, because of the presence of two types of new cell aggregates. The first was perivascular accumulation of cells. The second was cell aggregates that were not associated with blood vessels but located in areas with degraded or remodeled ECM. The expression of the proliferation marker Ki-67 was lower in normal aging ACLs and increased in both fibroblast- and chondrocyte-like cell aggregates in degenerated ACLs. This suggests that constitutive cell turnover declines with aging and that cell proliferation accounts at least in part for the formation of the cell aggregates in degenerated ACLs.

We used several differentiation (Sox9, Scx, and Runx2) and progenitor cell (α-SMA, STRO-1) markers the better to characterize ACL cell populations. Sox9 is a transcription factor involved in the modulation of the chondrocyte phenotype [28-30]. The percentage of Sox9-positive cells increased in the degenerated ACLs compared with normal and aging ACLs. In the degenerated ACLs, 60.1% of chondrocyte-like cells were Sox9 positive, whereas only 8.4% of fibroblast-like cells were Sox9 positive. These results suggest that a subpopulation of ACL cells differentiate into the chondrocyte-like phenotype in OA-affected knee joints. Transcription factor Scx is a distinct marker for tendon and ligament progenitors and for differentiated cells [20,31]. Scx promotes the expression of tenomodulin, a differentiation marker of tenocytes [32]. Normal human ACLs contain only a few Scx-positive cells. In degenerated ACLs, however, 50.1% of cells in the chondrocyte-like cell aggregates were positive for Scx, whereas only 3.4% of cells in the fibroblast-like cell aggregates were positive. Runx2, a regulator of chondrocyte hypertrophy, also was most strongly upregulated in the chondrocyte-like cell aggregates. Collectively, these results on differentiation markers suggest that chondrocyte-like aggregates contain abnormally differentiated cells that express both hypertrophic chondrocyte and immature tendon cell markers.

We used several markers that characterize progenitor cells and fibroblast subpopulations. Small populations of fibroblast-like cells are present in the circulation. In the adult, these cells arise mainly in the bone marrow [33,34] and resemble mesenchymal stem cells [35,36]. A second population also arises from adult bone and circulates as a monocyte-like cell that continues to express hematopoietic antigens such as CD34 and CD45. These cells also home to wound sites where they assume a fibroblast-like phenotype and produce collagens, containing vimentin cytoplasmic filaments. These cells have been termed fibrocytes [37]. Myofibroblasts are a subset of fibroblasts defined by the presence of organized α-SMA cytoplasmic filaments [38,39]. These cells have the potential to contract ECM, and they are enriched in sites of tissue injury and wound healing [40]. In degenerated ACLs, we found an increased number of fibroblast-like cells near the blood vessels. Many of these cells were STRO-1 positive and CD45 positive (data not shown). This result suggests that ACL degeneration is associated with recruitment of bone marrow-derived fibrocytes. In addition, chondrocyte-like cell aggregates also expressed STRO-1 and α-SMA. As these cells are more strongly positive for Ki-67 and located at a greater distance from blood vessels, they may originate from resident progenitors, which are present in normal ACLs [10,12]

Thus, ACL degeneration is associated with cell recruitment or proliferation, including progenitor cells or myofibroblasts. Chondrocyte-like cells in the degenerated ACLs might be abnormally differentiated from resident progenitor cells and locally proliferated, whereas fibroblast-like cells in the perivascular area might be bone marrow-derived cells.

To characterize ECM remodeling, we analyzed MMP-1, MMP-3, MMP-13, and several ECM molecules. The average percentage of MMP-1-positive cells, MMP-3-positive cells, and MMP-13 positive cells in ACLs from normal knees decreased with aging, but increased in degenerated ACLs from knees with severe cartilage degeneration. Cells expressing MMP-1, MMP-3, and MMP-13 in degenerated ACLs were predominantly cell aggregates of chondrocyte-like cells but not fibroblast-like cells. These results suggest that the decrease of MMP-1-, MMP-3-, and MMP-13-positive cells and total cell number density with aging may reflect a reduced capacity to remodel and maintain the tissue, whereas increased MMP-1-positive cells, MMP-3-positive cells, and MMP-13-positive cells in ACLs from knees with severe cartilage degeneration may be caused by phenotypic changes and contribute to degeneration.

We found reduced type I collagen around the chondrocyte-like cells in degenerated ACLs. Interestingly, type II, III, and X collagen and aggrecan were increased around these cells in degenerated ACLs. These results suggest that chondroid metaplasia contributes to the abnormal ECM production, which can lead to biomechanical failure [5,41].

## Conclusions

The relatively large sample set in this study showed the cellular changes and associated ECM changes. ACL aging is characterized by reduced cell density and activation. In contrast, ACL degeneration is associated with cell recruitment or proliferation, including progenitor cells or myofibroblasts. This cellular response to ACL tissue damage illustrates the regenerative capacity of ACL cells. Conversely, the chondrocyte-like phenotype produces abnormal ECM and may predispose to mechanical failure.

## Abbreviations

α-SMA: α-Smooth muscle actin; ACL: anterior cruciate ligament; ANOVA: analysis of variance; DSCF: Dwass-Steel-Critchlow-Fligner; DMEM; Dulbecco modified Eagle medium; ECM: extracellular matrix; FBS: fetal bovine serum; H&E: hematoxylin and eosin; ICRS: International Cartilage Repair Society; MMP-1: matrix metalloproteinase-1; MMP-3: matrix metalloproteinase-3; MMP-13: matrix metalloproteinase-13; OA: osteoarthritis; P1: passage 1; PBS: phosphate-buffered saline; Runx2: runt-related transcription factor 2; SEM: standard error of the mean.

## Competing interests

The authors declare that they have no competing interests.

## Authors' contributions

ML, HA, MK, and AH conceived of the study and participated in its design and coordination. AH and HN carried out histology and immunohistochemistry experiments and performed quantitative analysis. JK performed data analysis. All authors read and approved the final manuscript. Dr. Lotz had full access to all of the data in the study and takes responsibility for the integrity of the data and the accuracy of the data analysis.
